# Supplementary material for: Genome-wide gene-based analyses of weight loss interventions identify a potential role for NKX6.3 in metabolism
Source: Nat Commun. 2019 Feb 1;10:540. doi: 10.1038/s41467-019-08492-8 (PMC6358625; doi:10.1038/s41467-019-08492-8)
Supplement: Supplementary file 1 — Supplementary Information [file 41467_2019_8492_MOESM1_ESM.pdf]

Genome-wide gene-based analyses of weight loss interventions identify a potential role for NKX6.3 in metabolism

Valsesia et al.

Supplementary Figure 1: Population structure of the two weight loss cohorts

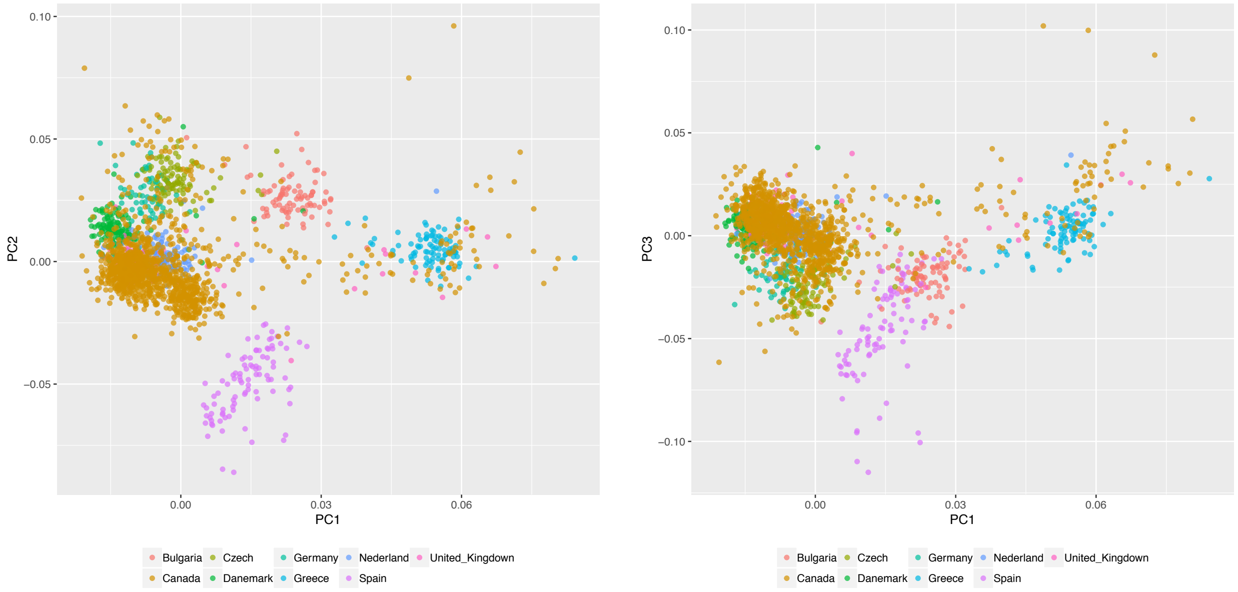

Explained variance is as follows: PC1=0.21%, PC2=0.09%, PC3=0.07%. Source data are provided as a Source Data file.

Supplementary Figure 2: *Ank/ANK1* has no effect on TAG level in *Drosophila*

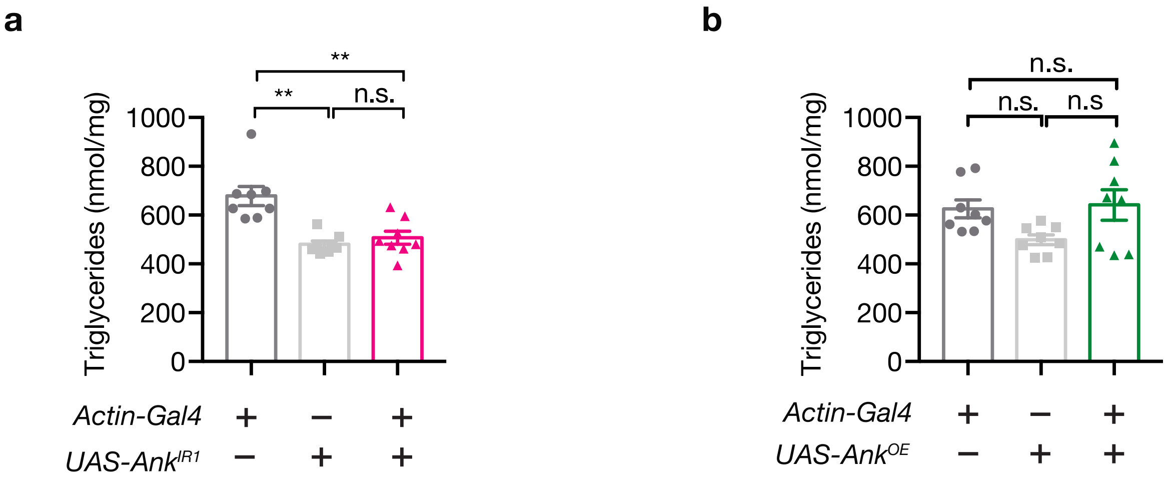

a, Whole-body *Ank/ANK1* RNAi did not affect TAG level in adult flies when compared to parental controls, n=8 groups, 5 flies each. **b**, TAG levels in whole-body *Ank/ANK1* over-expression animals compared to parental controls, n=8 groups, 5 flies each. Data are represented as means ± SEM. One-way ANOVA with Bonferroni's multiple comparisons test. **, $p<0.01$, n.s., not significant. Source data are provided as a Source Data file.

Supplementary Figure 3: Functional validation of HGTX/NKX6.3 in Drosophila

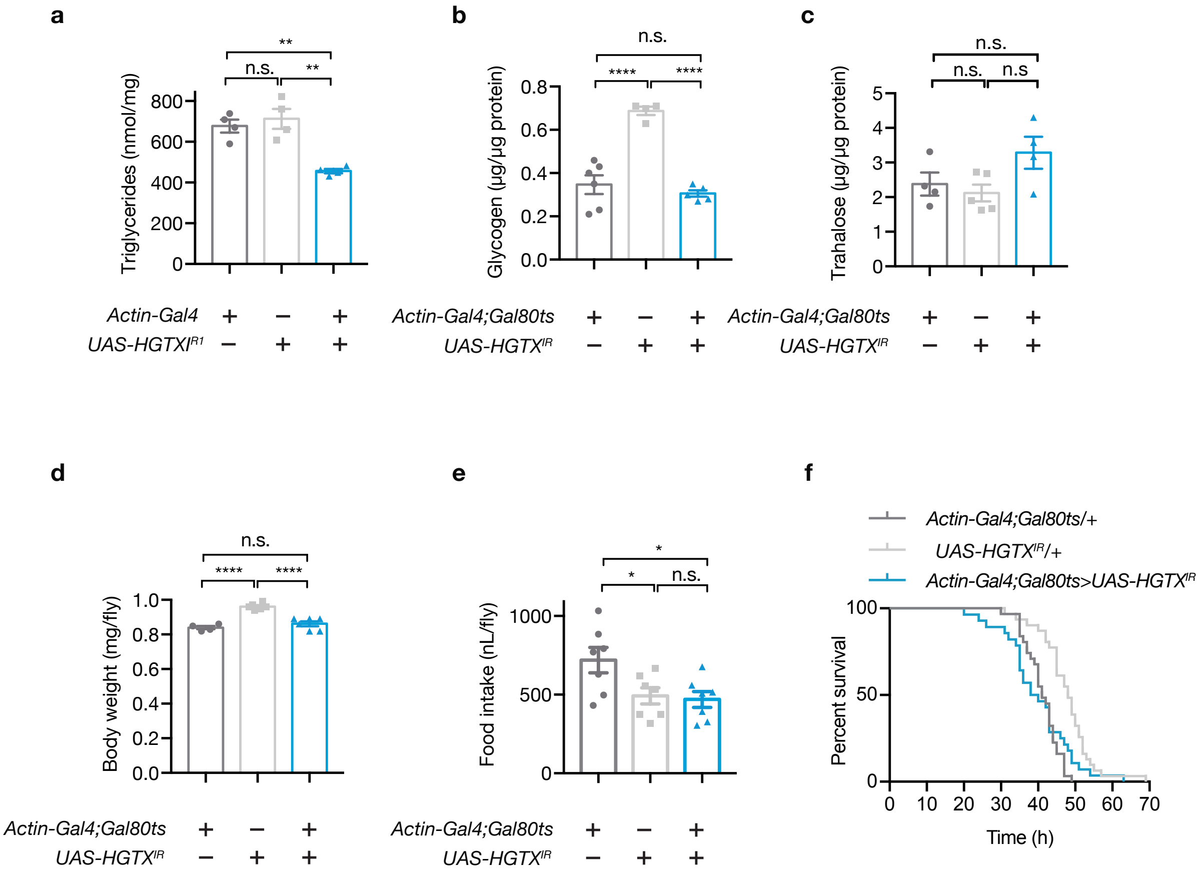

a TAG was reduced in adult inducible of whole-body *HGTX/NKX6.3* RNAi flies, n=4 groups, 5 flies each. **b-f** Metabolic phenotypes of glycogen (**b**, n=4-6 groups, 5 flies each), trehalose (**c**, n=4-5 groups, 5 flies each), body weight (**d**, n=4-6 groups, 10 flies each), food intake (**e**, n=7 groups, 5 flies each) and starvation (**f**, n=29-32 flies) were not significantly altered in adult inducible whole-body *HGTX/NKX6.3* RNAi flies. Data are represented as means ± SEM. One-way ANOVA with Bonferroni's multiple comparisons test. *, p<0.05, **, p<0.01, ***, p<0.001, ****, p<0.0001, n.s., not significant. Source data are provided as a Source Data file.

Supplementary Figure 4: Insulin-like peptides levels upon HTGX RNAi assays

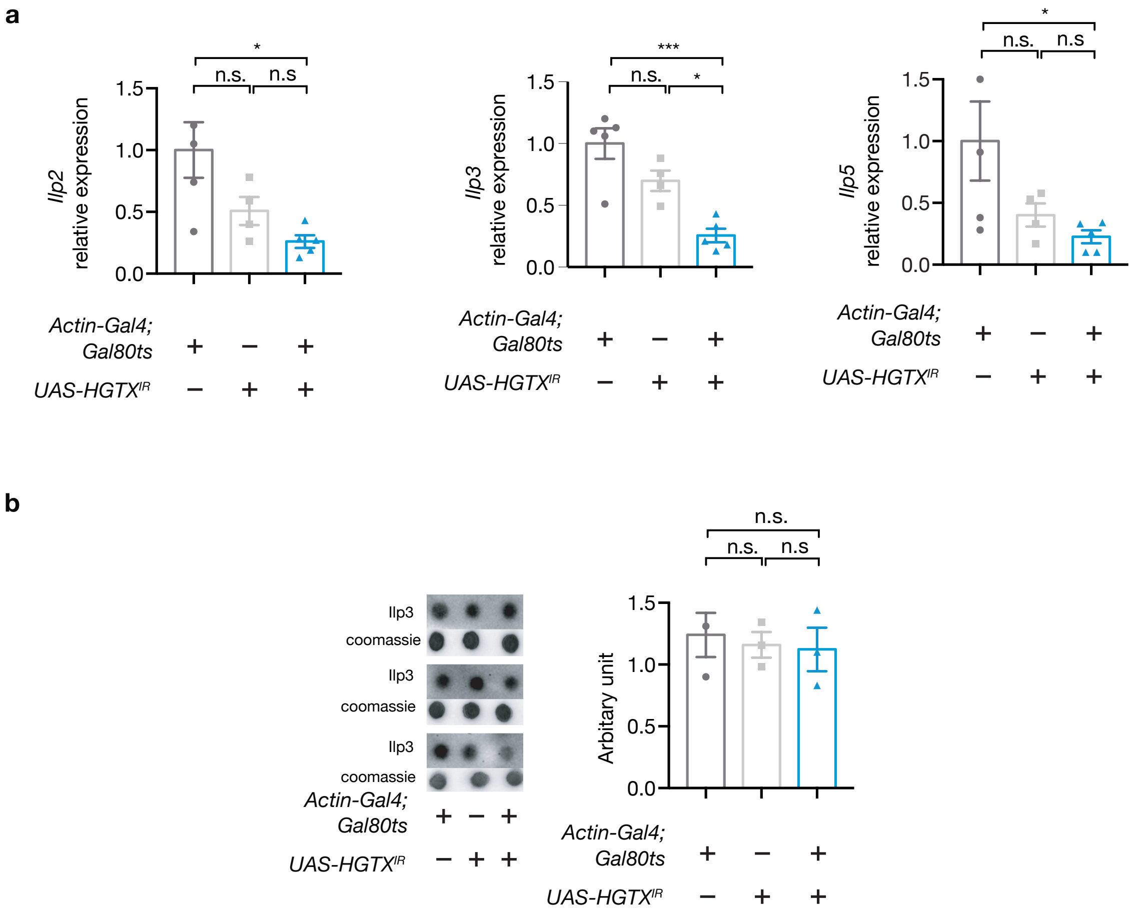

a, mRNA levels for insulin-like peptides 2, 3 and 5 in adult inducible *Gal4; Gal80ts>UAS-HGTX* RNAi flies, n=4-5 groups, 5 flies each. **b**, dot blot of Ilp3 protein in adult Gal4; Gal80ts>UAS-HGTX RNAi flies, Coomassie blue staining was used as loading control, density was quantified using image J, the bar graph represents the ratio of density from dot-blot vs Coomassie blue staining, n=3 biological replicates. Data are represented as means ± SEM. One-way ANOVA with Bonferroni's multiple comparisons test. n.s., not significant. Source data are provided as a Source Data file.

Supplementary Figure 5: Insulin-like peptides levels upon HTGX OE assays

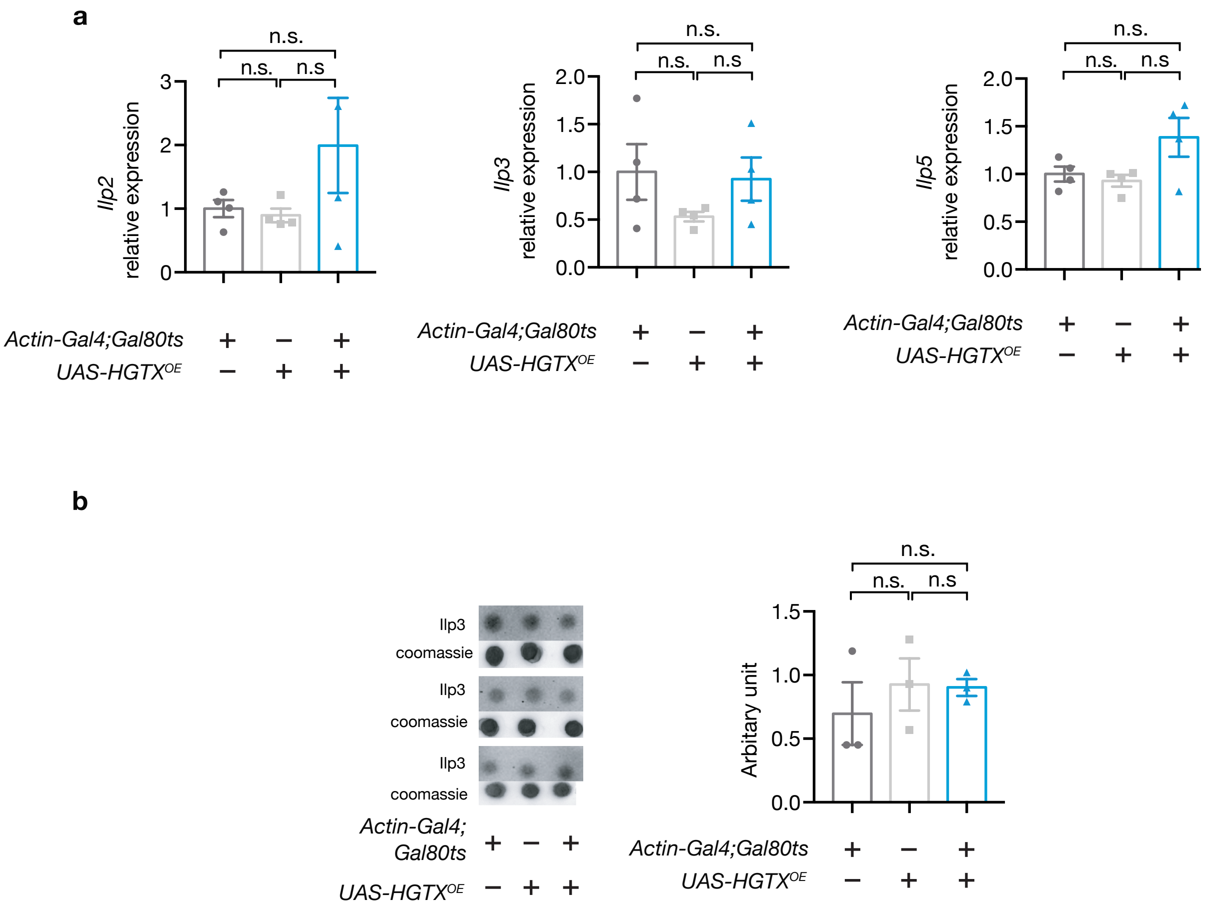

a, mRNA levels for insulin-like peptides 2, 3 and 5 in adult inducible *Gal4; Gal80ts>UAS-HGTX* OE flies, n=4 groups, 5 flies each. **b**, dot blot of Ilp3 protein in adult Gal4; Gal80ts>UAS-HGTX OE flies, Coomassie blue staining was used as loading control, density was quantified using image J, the bar graph represents the ratio of density from dot-blot vs Coomassie blue staining, n=3 biological replicates. Data are represented as means ± SEM. One-way ANOVA with Bonferroni's multiple comparisons test. n.s., not significant. Source data are provided as a Source Data file.
