# Supplementary material for: Genome-wide gene-based analyses of weight loss interventions identify a potential role for NKX6.3 in metabolism
Source: Nat Commun. 2019 Feb 1;10:540. doi: 10.1038/s41467-019-08492-8 (PMC6358625; doi:10.1038/s41467-019-08492-8)
Supplement: Supplementary file 3 — Reporting Summary [file 41467_2019_8492_MOESM3_ESM.pdf]

natureresearch

Corresponding author(s): Armand Valsesia

Reporting Summary

Nature Research wishes to improve the reproducibility of the work that we publish. This form provides structure for consistency and transparency in reporting. For further information on Nature Research policies, see Authors & Referees and the Editorial Policy Checklist.

Statistical parameters

When statistical analyses are reported, confirm that the following items are present in the relevant location (e.g. figure legend, table legend, main text, or Methods section).

n/a	Confirmed	
☐	☒	The exact sample size (*n*) for each experimental group/condition, given as a discrete number and unit of measurement
☐	☒	An indication of whether measurements were taken from distinct samples or whether the same sample was measured repeatedly
☐	☒	The statistical test(s) used AND whether they are one- or two-sided<br>*Only common tests should be described solely by name; describe more complex techniques in the Methods section.*
☐	☒	A description of all covariates tested
☐	☒	A description of any assumptions or corrections, such as tests of normality and adjustment for multiple comparisons
☐	☒	A full description of the statistics including central tendency (e.g. means) or other basic estimates (e.g. regression coefficient) AND variation (e.g. standard deviation) or associated estimates of uncertainty (e.g. confidence intervals)
☐	☒	For null hypothesis testing, the test statistic (e.g. *F*, *t*, *r*) with confidence intervals, effect sizes, degrees of freedom and *P* value noted<br>*Give P values as exact values whenever suitable.*
☒	☐	For Bayesian analysis, information on the choice of priors and Markov chain Monte Carlo settings
☒	☐	For hierarchical and complex designs, identification of the appropriate level for tests and full reporting of outcomes
☐	☒	Estimates of effect sizes (e.g. Cohen's *d*, Pearson's *r*), indicating how they were calculated
☐	☒	Clearly defined error bars<br>*State explicitly what error bars represent (e.g. SD, SE, CI)*

Our web collection on statistics for biologists may be useful.

Software and code

Policy information about availability of computer code

Data collection

Genotype data were generated using HumanCoreExome-12 v1.1 with 264,909 tag SNP marker and 244,593 exome-focused markers. They were processed with the Illumina TM platform following Infinium® HD Assay Ultra, Manual according to manufacturer's instructions. Genotypes were called with the GenomeStudio Software (Illumina). Quality control procedure followed recommendations from the GenABEL package67 and excluded SNPs with call rate < 95%, violating Hardy-Weinberg equilibrium (FDR<20%), low minor allele frequency < 1%. Subjects were excluded if they had low call rate (<95%), abnormally high autosomal heterozygosity (FDR<1%), an XXY karyotype, or gender inconsistencies between genotype data and clinical records. For subjects with high identity-by-state (IBS>95%), only the one having the highest call rate was kept. Principal component analyses (PCA) were performed independently on each cohort to discard subjects that were outliers in term of genetic structure. Subjects from both cohorts were all of European ancestry and the two cohorts had similar genetic structure (Figure S1). Upon all genetic QCs, 1166 Ottawa and 789 DiOGenes subjects were kept for subsequent analyses.
Genotype imputation was then performed using SHAPEIT68 and IMPUTE269 based on the European reference panel from the 1000 Genome project70 (March 2012 release, phase 1 version 3). Imputation post-filtering removed SNPs with reference allele frequency less than 1% and INFO score < 0.8. Upon such filtering, data for 4.9M imputed SNPs were available for both datasets.

Functional analyses in Fly
Fly strains. Fly stocks were maintained on standard diet with agar, sugar and yeast and were raised in 25°C incubator at a 12/12 dark and night cycle. Actin-Gal4 and TubGal80ts was from Bloomington. UAS-AnkIR (GD25945), UAS-HGTXIR1(GD12608), UAS-HGTXIR2 (KK109732)， UAS-CG3209 IR2 (KK10281) were from the VDRC.
Triglyceride assay: 5 male flies were weighted and homogenised in 200 µl PBST (PBS + 0.05% Tween 20) on ice, then sonicated for 10s

using a probe sonicator on ice. After sonication, 800µl ice-cold PBST was added and mixed thoroughly. 50 µl of the mixture was used to determine the triglycerides using the Roche triglycerides kit (11730711216) under the manufacturer's instructions, and 10 µl of the mixture was used to determine to protein using Bradford protein assay kit (Sigma). Triglycerides were normalized to protein level.
Glycogen assay: Glycogen was determined by measuring the glucose degraded from glycogen using amyoglucosidases. 5 male flies were homogenized and dissolved in 1M KOH solutions. After twice 95% ethanol extraction, the pellet was resuspended 1ml amyoglucosidase reaction buffer (0.3 mg/ml in 0.25 M acetate buffer, pH 4.75) and incubated in the 37°C shaking incubator overnight and a glucose assay was performed.
Trehalose assay: Trehalose was determined by measuring the glucose degraded from trehalose using trehalosase. 20 male flies' heads were homogenized in 200 µl ice-cold TB buffer (5 mM Tris pH 6.6, 136 mM NaCl, 2.7 mM KCL), then centrifuged at 4 ºC for 5 min at maximum speed and collect the supernatant. 20 ul of supernatant was used to measure glucose and protein, respectively. 50 µl of supernatant was mixed with 50 µl TE solution (10 µl trehalose in 1.5 ml TB buffer). Incubated at 37°C for overnight. Centrifuged at 4 °C for max speed for 3 min and then 20 ul of supernatant was used to measure glucose assay. Glucose assay: 50 µl of mixture was used to determine the [glucose] using Thermo Infinite Glucose kit (TR15421) as the manufacturer's instructions.
Body weight
Body weight were measured by analytic balance
Food intake assay
Food intake was measured by CAFE assay. Food intake was measured over 24 hours. Empty vials were used for evaporation controls. All food intake experiments were set up at Zeitgeber time 6–8, and food intake was recorded exactly, 24 hours after start of food loading.
Starvation assay
Male flies were put into DAM2 tubes with 2% agar and the death was monitored per hour.
qPCR

1µg of mRNA was reversely transcribed into cDNA using SuperScript® III First-Strand Synthesis System (Invitrogen). All primers used for qPCR that have been prescreened for efficiency and specificity. RT-PCR was performed using SensimixTM probe kit (Bioline). The program is following: 95°C 10 min, 40 cycles of 95°C, 15s; 55°C, 15s; 72°C, 15s. The reactions were run on Light Cycle® 480 (Roche). The gene expression was normalized to the reference gene rp49. The following primers are used:
HGTX forward: CGAGTCGCAGGTTAAGGTCT;
HGTX Reverse: CCGCCCATATCGTCCTGTTT;
Rp49 forward: CGGATCGATATGCTAAGCTGT;
Rp49 reverse: GCGCTTGTTCGATCCGTA;
Ilp2 forward: TGAGTATGGTGTGCGAGG;
Ilp2 Reverse: CTCTCCACGATTCCTTGC;
Ilp3 forward: GAACTTTGGACCCCGTGAA;
Ilp3 Reverse: TGAGCATCTGAACCGAACT;
Ilp5 forward: CAAACGAGGCACCTTGGG;
Ilp5 Reverse: AGCTATCCAAATCCGCCA;

Data analysis

Single-SNP analyses were performed using BOLT-LMM24, a Bayesian linear mixed effect model to adjust for population structure and cryptic relatedness between individuals. The rate of weight loss was adjusted for sex, age and starting BMI. Results from the two cohorts, were subsequently meta-analyzed using Genome-Wide Association Meta Analysis (GWAMA) software71 with random-effect modeling and a double genomic-control (GC) correction72 (GC correction at study-level and also at meta-analysis level).
Gene-based analysis was performed using the VEGAS (versatile gene-based association study) approach20. This method integrates single-SNP p-values at the gene-level and accounts for LD patterns through Monte-Carlo simulations. Analysis was made with the following settings: European ancestry population from the 1000 Genome project as reference population for LD patterns, set-based test using the top 80% SNPs and a gene block size set to 20kb. Such setting assigns to each gene, its neighboring SNPs (within 20kb) and discard the 20% SNPs having the least significant p-value. For regions with closely located genes (e.g. gene clusters), potentially the same SNPs could be assign to different genes, leading to different "gene-locus" having similar p-values. This is not an issue as the goal of such analyses is only to highlight loci associated with the trait of interest. Adjustment for multiple-testing was performed using the Benjamini-Hochberg correction73. Genome-wide significance threshold was set to FDR < 5%.
Prioritization of GWA signals was performed using a Bayesian framework to model the joint likelihood of association p-values with large-scale epigenomic annotations. Such risk variance inference was performed using the RiVIERA-beta framework21 and with 450 epigenomic annotations (including histone marks, DNase I hypersensitivity, transcription factor binding, and localization within exons). The goal of this framework is to infer for each input SNP the posterior probability of disease given its association p-value and overlap in functional annotations. Epigenomic annotations were retrieved from Pickrell et al.74.
Analysis of fly phenotypes was performed using one-way ANOVA with Bonferroni adjustment for multiple comparisons.

For manuscripts utilizing custom algorithms or software that are central to the research but not yet described in published literature, software must be made available to editors/reviewers upon request. We strongly encourage code deposition in a community repository (e.g. GitHub). See the Nature Research guidelines for submitting code & software for further information.

Data

Policy information about availability of data

All manuscripts must include a data availability statement. This statement should provide the following information, where applicable:

- Accession codes, unique identifiers, or web links for publicly available datasets
- A list of figures that have associated raw data
- A description of any restrictions on data availability

The data that support the findings of this study are available from the corresponding author (Armand.Valsesia@rd.nestle.com) upon reasonable request.

Field-specific reporting

Please select the best fit for your research. If you are not sure, read the appropriate sections before making your selection.

☒ Life sciences ☐ Behavioural & social sciences ☐ Ecological, evolutionary & environmental sciences

For a reference copy of the document with all sections, see nature.com/authors/policies/ReportingSummary-flat.pdf

Life sciences study design

All studies must disclose on these points even when the disclosure is negative.

Sample size	These analyses are exploratory and no sample size computation could be performed.<br><br>The Canadian samples consisted of patients enrolled in the Weight Management Clinic16 who had completed 6 to 12 weeks meal-replacement regimen consisting of a product uniquely available in Canada, Optifast900 (Nestlé Health Science, Switzerland). Program adherence criteria included: attendance to 75% of clinical sessions and strict adherence to the meal replacement (evaluated by the number of meal-replacement products that were consumed). Patients under medication known to affect rate of weight loss or glucose homeostasis or abnormal thyroid indices were excluded from the analyses. In total, 1436 Optifast900 subjects were eligible for genotyping and had available weight measurements.<br>The DiOGenes study is an interventional, multi-center pan-European study17,18. Eight partners participated to the study: Bulgaria, Czech Republic, Denmark, Germany, Greece, the Netherlands, Spain and United Kingdom. Participants followed an 8-week LCD. The LCD provided 800 kcal per day with the use of a meal-replacement product (Modifast, Nutrition et Santé France). Participants could also eat up to 400g of vegetables (corresponding to a maximal addition of 200kcal/day). In total, 888 DiOGenes subjects were included for genotyping. The macronutrient composition of the two meal-replacement products was considered similar (fat=17% and14%; proteins=34% and 42% and carbohydrates=49% and 44%, respectively for the Optifast and Modifast products).
Data exclusions	Data inclusions/exclusions were based on availability of weight measures (at baseline and upon 5-weeks of low-caloric diet intervention) and whether genotyping led to high quality data.<br>Genotyping QC was performed as follows:<br>Quality control procedure followed recommendations from the GenABEL package67 and excluded SNPs with call rate < 95%, violating Hardy-Weinberg equilibrium (FDR<20%), low minor allele frequency < 1%. Subjects were excluded if they had low call rate (<95%), abnormally high autosomal heterozygosity (FDR<1%), an XXY karyotype, or gender inconsistencies between genotype data and clinical records. For subjects with high identity-by-state (IBS>95%), only the one having the highest call rate was kept. Principal component analyses (PCA) were performed independently on each cohort to discard subjects that were outliers in term of genetic structure. Subjects from both cohorts were all of European ancestry and the two cohorts had similar genetic structure (Figure S1). Upon all genetic QCs, 1166 Ottawa and 789 DiOGenes subjects were kept for subsequent analyses.
Replication	Discovery analyses were performed using the Canadian cohort.<br>Replication was performed using the DiOGenes cohort.
Randomization	Subjects from a same cohort were given the same meal-replacement product (Optifast900 for the Canadian cohort; Modifast800 for the DiOGenes cohort).<br><br>Genotyping was performed using a complete randomization design. The (randomly attributed) plate ID was then checked for possible association with gender, BMI, rate of weight loss, glycemia and countries. Subsequent analyses (using genotyped data) did not reveal any batch effects linked with the design, the aforementioned co-variates, or genetic ancestry of the participants
Blinding	No blinding was used. All participants from the same cohort followed the same intervention.

Reporting for specific materials, systems and methods

Materials & experimental systems

n/a	Involved in the study
☒	☐ Unique biological materials
☐	☒ Antibodies
☒	☐ Eukaryotic cell lines
☒	☐ Palaeontology
☐	☒ Animals and other organisms
☐	☒ Human research participants

Methods

n/a	Involved in the study
☒	☐ ChIP-seq
☒	☐ Flow cytometry
☒	☐ MRI-based neuroimaging

Antibodies

Antibodies used	rabbit-anti-Ilp3 antibody,source: Dr. Jan Veenstra, Bordeaux University France
Validation	Veenstra, J. A., Agricola, H.-J. & Sellami, A. Regulatory peptides in fruit fly midgut. Cell Tissue Res. 334, 499–516 (2008).<br>Kim, J. & Neufeld, T. P. Dietary sugar promotes systemic TOR activation in Drosophila through AKH-dependent selective secretion of Dilp3. Nat. Commun. 6, 6846 (2015).

Animals and other organisms

Policy information about studies involving animals; ARRIVE guidelines recommended for reporting animal research

Laboratory animals	Drosophila melanogaster strain were as follows: UAS-AnkIR (GD25945), UAS-HGTXIR1(GD12608), UAS-HGTXIR2 (KK109732)， UAS-CG3209 IR2 (KK10281) were from the VDRC.<br>Only male flies with 4-7 days or 10-13 days were used for experiments.
Wild animals	NA
Field-collected samples	NA

Human research participants

Policy information about studies involving human research participants

Population characteristics	Population characteristics are detailed in table 1<br><br>And the results section include the following paragraph:<br>The Optifast900 cohort included both obese and severely obese subjects (mean BMI =43.2 kg/m2 +/- 0.3 standard error of the mean) and the DiOGenes cohort included overweight and obese participants (mean BMI=34.5kg/m2 +/- 0.2). Clinical characteristics of the participants are available in Table 1. Upon a 5-week low calorie diet (LCD), participants lost on average 9.3% (11.3kg) and 7.5% (7.5kg) of initial body weight, respectively for the Optifast900 and DiOGenes participants. At baseline, Optifast900 participants were considered more insulin-resistant than DiOGenes subjects (HOMA-IR=4.16 +/- 0.14 vs. 3.15 +/- 0.10), as expected given the more severe obesity.
Recruitment	Ottawa participants are obese subjects referred (by their doctor) to an obesity clinic for a weight loss intervention.<br>Diogenes participants are overweight/obese non-diabetic subjects that responded to recruitment criteria of the Diogenes weight maintenance study: https://clinicaltrials.gov/ct2/show/NCT00390637
