# Supplementary material for: Genome-wide gene-based analyses of weight loss interventions identify a potential role for NKX6.3 in metabolism
Source: Nat Commun. 2019 Feb 1;10:540. doi: 10.1038/s41467-019-08492-8 (PMC6358625; doi:10.1038/s41467-019-08492-8)
Supplement: Supplementary file 4 — Description of Additional Supplementary Files [file 41467_2019_8492_MOESM4_ESM.pdf]

Supplementary Data 1

Gene-based association summary statistics from the Ottawa cohort (discovery)

Supplementary Data 2

Gene-based association summary statistics from the DiOGenes cohort (replication)

Supplementary Data 3

Gene-based association summary statistics from meta-analysis of the Ottawa and DiOGenes cohorts

Supplementary Data 4

SNP-based association summary statistics from the Ottawa cohort (discovery)

Supplementary Data 5

SNP -based association summary statistics from the DiOGenes cohort (replication)

Supplementary Data 6

SNP -based association summary statistics from meta-analysis of the Ottawa and DiOGenes cohorts
